# *FLOWERING LOCUS C *-dependent and -independent regulation of the circadian clock by the autonomous and vernalization pathways

**DOI:** 10.1186/1471-2229-6-10

**Published:** 2006-05-31

**Authors:** Neeraj Salathia, Seth J Davis, James R Lynn, Scott D Michaels, Richard M Amasino, Andrew J Millar

**Affiliations:** 1 Department of Biological Sciences, University of Warwick, Coventry CV4 7AL, UK; 2 Warwick HRI, Wellesbourne, Warwick CV35 9EF, UK; 3 Department of Biochemistry, University of Wisconsin-Madison, Madison, WI 53706, USA; 4 Bauer Center for Genomics Research, Harvard University, Cambridge, MA 02138, USA; 5 Department of Plant Developmental Biology, Max Planck Institute for Plant Breeding Research, Cologne 50829, Germany; 6 Department of Biology, Indiana University, Bloomington, IN 47405, USA; 7 School of Biological Sciences, Edinburgh University, Edinburgh EH9 3JH, UK

## Abstract

**Background:**

The circadian system drives pervasive biological rhythms in plants. Circadian clocks integrate endogenous timing information with environmental signals, in order to match rhythmic outputs to the local day/night cycle. Multiple signaling pathways affect the circadian system, in ways that are likely to be adaptively significant. Our previous studies of natural genetic variation in *Arabidopsis thaliana *accessions implicated *FLOWERING LOCUS C *(*FLC*) as a circadian-clock regulator. The MADS-box transcription factor *FLC *is best known as a regulator of flowering time. Its activity is regulated by many regulatory genes in the "autonomous" and vernalization-dependent flowering pathways. We tested whether these same pathways affect the circadian system.

**Results:**

Genes in the autonomous flowering pathway, including *FLC*, were found to regulate circadian period in Arabidopsis. The mechanisms involved are similar, but not identical, to the control of flowering time. By mutant analyses, we demonstrate a graded effect of *FLC *expression upon circadian period. Related MADS-box genes had less effect on clock function. We also reveal an unexpected vernalization-dependent alteration of periodicity.

**Conclusion:**

This study has aided in the understanding of *FLC*'s role in the clock, as it reveals that the network affecting circadian timing is partially overlapping with the floral-regulatory network. We also show a link between vernalization and circadian period. This finding may be of ecological relevance for developmental programing in other plant species.

## Background

Most eukaryotes and some prokaryotes have evolved a circadian clock to adapt to the 24 h day/night cycle. These clocks drive biological rhythms in many aspects of metabolism, physiology, and behavior, all with a period close to 24 h [1]. Circadian rhythms affect fundamental processes of plant life, such as photosynthesis and cell elongation [2]. Day-length measurement (photoperiodism) also depends on the circadian clock, which thereby controls seasonal rhythms such as the timing of flowering [3,4]. Described molecular-genetic models from various circadian organisms has each included a gene circuit with negative-feedback elements, involving 24 h rhythms in the levels of positively- and negatively-acting transcriptional regulators [5]. In Arabidopsis, there is emerging evidence that a set of about 20 genes create one, or more, feedback circuits (the 'circadian oscillator') to generate the 24 h period [2,6], and this rhythmically regulates the level of around 6% of transcripts [7].

Circadian clocks, including those of Arabidopsis, are reset by light and temperature signals in a characteristic fashion that entrains the clock to the local time in its environment [6]. However, circadian period is buffered against long-term changes in temperature, such that the period remains close to 24 h when assayed at various constant temperatures, over a physiologically relevant range. Such 'temperature compensation' is another distinguishing feature of circadian clocks, including those in Arabidopsis [8,9]. Whereas the mechanisms of photic entrainment are being elucidated [2,6], those governing temperature entrainment and temperature compensation remain to be determined.

A circadian clock maintains accurate timing because it is buffered against many environmental changes, yet several environmental-signaling pathways must affect the circadian oscillator for entrainment to occur. Limiting the input connections to the circadian clock from the rest of the plant-signaling network provides a potential mechanism to balance the opposing requirements of homeostasis and entrainment. In the gene network that regulates flowering time, for example, the circadian clock is an integral part of the photoperiodic sensor, receiving input from light signaling [3,4]. Current models indicate that output from the photoperiod pathway converges with several other pathways that control flowering time, but the photoperiod sensor is thought to receive no input from those pathways [3,4]. Genetic variation among Arabidopsis accessions prompted us to reexamine this notion.

Substantial natural variation has been detected in clock-affecting genes, based upon assays of rhythmic leaf movement under constant light [9-11]. This assay allowed us to map Quantitative Trait Loci (QTL) that affect circadian period in recombinant populations derived from crosses between accessions Cape Verde Islands (Cvi) × Landsberg *erecta *(L*er*) and Columbia (Col) × L*er *[10]. In each population, we located a major QTL towards the top of chromosome 5, close to the map location of *FLOWERING LOCUS C *(*FLC*). The L*er *allele of *FLC *is weakly functional due to a transposon insertion within an intron of *FLC *[12,13]. The populations that we used included L*er *as one parent, therefore *FLC *function segregates in these recombinant populations [14]. The L*er *allele of the QTL shortened the circadian period by 0.8 h, as did independent *flc *mutant alleles, leading us to conclude that the known allelic variation in *FLC *could account for the QTL [10].

*FLC *encodes a MADS-box transcription factor that had no known function in the circadian clock, but was well-characterized as a repressor of flowering. *FLC *expression is enhanced by *FRIGIDA *(*FRI*) and its paralogues, which are active in many late-flowering accessions [12,13]. *FLC *transcription is suppressed by genes of the autonomous floral-promotion pathways and by prolonged cold temperatures (vernalization, indicative of winter in nature; reviewed in [3,11,12]). As *flc *mutants harbor an altered circadian period, we hypothesized that other genetic and physiological regulators of *FLC *would have predictable effects on the circadian clock. We therefore tested whether the network of *FLC *regulators that was defined with respect to flowering time also functions in the control of circadian period. A substantial number of upstream regulators, a related gene, and a downstream target gene do have similar functions, but we also find clear distinctions between *FLC*-related pathways. The circadian period is also sensitive to vernalization, revealing a previously-unrecognized connection between the gene circuits involved in responses to daily and to seasonal rhythms.

## Results

### Dose-dependent effect of *FLC *on circadian period

**Figure 1:**
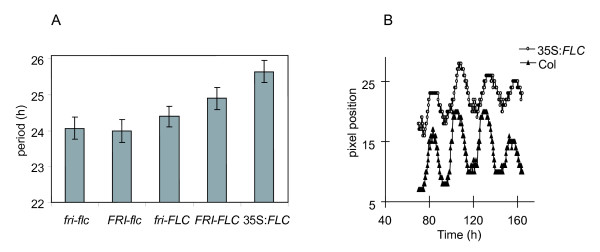
**Effects of null *flc *and 35S: *FLC*on circadian period **1a) Circadian periods of *FRI-FLC *mutant combination and 35S:*FLC *plants assayed by rhythmic leaf movement in constant dim white light for approx. 1 week (*n *= 36–155). *fri-flc *τ = 24.07 ± 0.31 [SEM] h, *FRI-flc τ *= 24.00 ± 0.32 h, *fri-FLC τ *= 24.40 ± 0.29 h, *FRI-FLC *τ = 24.90 ± 0.31 h. 1b) Representative traces of rhythmic leaf movement of a 35S:*FLC *(white circles) and wild-type (filled triangles).

*FLC *RNA abundance correlates with repression of flowering time and quantitatively mediates the vernalization response of flowering time [15]. We sought to determine whether *FLC *expression levels similarly regulated the circadian clock. To assay the plant's endogenous circadian period, rhythmic movement of the primary leaves of Arabidopsis seedlings were measured by video imaging under constant white light. Relatively large numbers of plants were tested in replicate experiments in order to increase the sensitivity of the assays, allowing us to detect small changes in circadian period (see experimental procedures). *FLC *RNA abundance was manipulated by two methods. Firstly, we tested a line expressing *FLC *from the CaMV 35S promoter (*35S:FLC*). This transgenic construction strongly delays flowering time. The *35S:FLC *line had a circadian period lengthened by over 1 hour in multiple experiments (τ = 25.65 ± 0.31 [SEM] h vs. 24.44 ± 0.17 h, *P *< 0.0001) (Figure 1, Table 1). Secondly, circadian experiments were carried out on lines harboring the four possible homozygous combinations of *FLC *alleles with alleles of the *FLC *activator, *FRIGIDA *(*FRI*), all uniformly in a Col background. These comprised the functional alleles*FRI-Sf2 *and *FLC-Col *(*FRI *and *FLC*) and recessive alleles *fri-Col *and *flc-3 *(*fri *and *flc) *[16]. Lines harboring functional *FLC *had a lengthened circadian period compared to *flc *lines (Figure 1). Joint statistical analysis of 6 replicate experiments was used to reveal that *FLC *alone increased circadian period by an average of 0.6 hours, across both *FRI *genotypes (*P *= 0.004, Figure 1, Table 1). This is in agreement with the results of Swarup *et al*. (1999) [10], who showed that *FLC *lengthened circadian period in both *FRI *and *fri *backgrounds. Our findings are also consistent with the function of *FLC *in flowering time. *fri;FLC *plants flower slightly later than *fri;flc *mutants under non-inductive photoperiods, indicating *FLC *can function independently of *FRI *in the floral pathway [17]. Increasing the abundance of *FLC *transcript is therefore sufficient to increase the circadian period of the plant. The greater effect of *35S:FLC *compared to endogenous *FLC *suggests that increasing *FLC *RNA levels increase circadian period in a graded manner, similar to *FLC*'s dose-dependent delay of flowering [16,18]. It might be possible that a broader spatial domain of *FLC *expression in *35S:FLC *plants contributed to this result, though such effects on the location of FLC expression have not previously been implicated in flowering-time control.

We suggest that functional *FRI *caused an additional increase in period in the presence of functional *FLC *(Figure 1; see also Figure 3). Although *FRI;FLC *had the longest mean period in each experiment, the interaction between *FRI *and *FLC *was not significant in the joint analysis (data not shown). This indicates that *FRI *can increase circadian period weakly and less consistently than *FLC*. In contrast, functional *FRI *strongly enhances *FLC *RNA levels and severely delays flowering [16,18], highlighting an obvious difference between circadian and flowering time control. Again, it is possible that *FRI *does not enhance *FLC *expression in the cells that regulate leaf movement as much as *35S:FLC*.

### Effects of *FLC *regulators on circadian period

**Figure 2:**
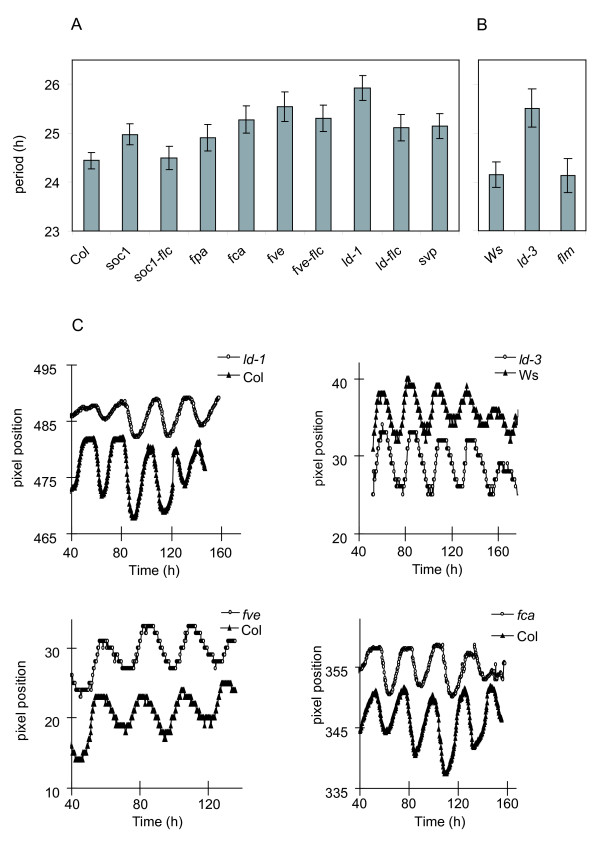
**Circadian period of autonomous flowering time pathway mutants **Circadian period estimates for mutants in the autonomous flowering-time pathway and their respective wild-types. Period was assayed by rhythmic leaf movement in constant white light for approx. 1 week (*n *= 25–155). 1a) genotypes in the Col-0 genetic background. 1b) Genotypes in the Ws-2 background. 1c) Representative leaf movement traces of Arabidopsis mutant (white circles) and wild-type seedlings (filled triangles), imaged in constant dim white light for approx. 1 week.

The above results lead us to the hypothesis that any factor that modulates *FLC *expression levels, including the genes of the autonomous pathway, would affect circadian period. In order to compare the network of circadian regulators to the pathways that regulates flowering time, we analyzed the circadian period of plants carrying mutations in several genes of the autonomous flowering-time pathway (Figure 2), all of which contribute to regulate *FLC *RNA abundance (reviewed in Henderson *et al*., 2003 [19]).

#### FCA

*FCA *is one of the genes that defines the autonomous flowering-time pathway. *FCA *encodes a nuclear-localized protein with RNA recognition motifs. It is involved in 3' RNA processing of *FCA *transcripts through physical interaction with the FY protein [20-22]. *FCA *promotes flowering by repression of *FLC *RNA levels [17,23]: plants with an *fca *lesion have high levels of *FLC *RNA and flower late. Assays on *fca *mutants revealed an increase in circadian period of nearly 1 hour compared to the Col-0 wild-type (τ = 25.28 ± 0.28 h vs. 24.44 ± 0.17 h, *P *= 0.002), in line with the hypothesis that elevated *FLC *levels increase circadian period (Figure 2 and 5, Table 2).

#### LUMINIDEPENDENS (LD)

*LD *represses *FLC *expression and encodes a predicted nuclear protein with a glutamine rich Carboxy terminus, suggesting a role as a transcriptional regulator [16,24,25]. Of the autonomous pathway genes tested, *LD *had the most striking effect on circadian period. A period increase of approximately 1.5 hours in *ld *mutants was observed relative to the wild type, in two genetic backgrounds (τ = 25.93 ± 0.25 h vs. 24.44 ± 0.17 h for *ld-1 *in a Col background, *P*= <0.0001 and τ = 25.51 ± 0.39 h vs. 24.15 ± 0.26 h for *ld-3 *in a Ws-2 background, *P*= 0.0012) (Figure 2 and 5, Table 2). In order to test whether the circadian effect of *LD *required *FLC*, we assayed the period of an *ld; flc *double mutant [17,23]. The *ld; flc *double mutant lines decreased circadian period by 0.8 h relative to the *ld *single mutant (τ = 25.12 ± 0.27 h vs. 25.93 ± 0.25 h, *P *= 0.005) (Figure 2). The *ld; flc *double mutant had a longer circadian period than wild-type plants (τ = 25.12 ± 0.27 h vs. 24.44 ± 0.17 h, *P *= 0.01) (Figure 2 and 5, Table 2), which in turn had a longer period than the *flc *mutants (Figure 1). Thus the *ld;flc *double mutant has an intermediate phenotype, with a significantly longer period than the *flc *single mutant but shorter than the *ld *single mutant. We conclude that the effect of *LD *on period is at most partly dependent on *FLC*, and*LD *function must also influence the circadian clock independently of *FLC*. This contrasts with the *FLC*-dependence of the effects of *LD *on flowering [17].

#### FVE

*FVE *encodes a component of a histone-acetylation complex, which functions to strongly suppresses *FLC *RNA abundance, to thus function in the flowering-time pathway [23,26,27]. Additionally, *FVE *affects morphological traits such as leaf shape and inflorescence patterns [28]. The *fve *mutant increased circadian period by over 1 hour (τ = 25.54 ± 0.30 h vs. 24.44 ± 0.17 h, *P *= 0.0002). To test whether this was due to an increase in *FLC *activity in the mutant, we assayed the *fve; flc *double mutant. We found a statistically insignificant decrease in circadian period (0.24 h) in the double mutant compared to the *fve *single mutant (τ = 25.30 ± 0.27 h vs. 25.54 ± 0.30 h, *P*= 0.45), so the period of the *fve; flc *double mutant retained a significantly lengthened period (Figure 2 and 5, Table 2). These results indicate to us that *FVE *regulates circadian timing in a manner that is largely independent of *FLC*, again in contrast to its effect on flowering [17].

#### FPA

*FPA *is predicted to encode a protein that contains RNA recognition motifs and also represses *FLC *RNA and function in the flowering-time pathway [29]. The *fpa *mutant showed only a small increase in period (0.47 h) compared to the wild type, and this modest effect was not statistically significant (τ = 24.91 ± 0.27 h vs 24.44 ± 0.17 h, *P *= 0.08) (Figure 2 and 5, Table 2). Therefore, *FPA *seems to have less effect on the circadian clock of Arabidopsis than the other mutants tested. Though *FPA *has been functionally linked with *FVE *in the flowering time network, it differs from *FVE *in that *FPA *is less sensitive to *FLC *gene dosage [23], *FPA *interacts with genes of the photoperiodic-response pathway [30], and has been implicated in the gibberellin response [31]. These differences may reflect a range of molecular functions that limits the effect of *FPA *on circadian timing.

### Effects of other MADS-box genes on circadian period

#### SUPPRESSOR OF OVEREXPRESSION OF CONSTANS 1 (SOC1)

*SOC1 *(also referred to as *AGL20*) is a MADS-box gene that activates flowering, in response to signals from the autonomous and photoperiodic flowering-time pathways [32,33]. Unlike the genes tested above, *SOC1 *is described as a target of *FLC*-mediated transcriptional repression in the flowering-time pathway [17]. The *soc1 *mutant has a small increase in circadian period relative to wild type (τ = 24.98 h ± 0.22 h vs. 24.44 ± 0.17 h, *P *= 0.008), indicating that *SOC1 *normally shortens circadian period (Figure 2 and 5, Table 2). If *FLC *represses *SOC1 *expression, this result is consistent with the idea that *flc *mutants shorten circadian period at least in part *via *increased expression of *SOC1*. We assayed the *soc1; flc *double mutant in order to test this notion, which would predict a long period in the *soc1; flc *double mutant, similar to the *soc1 *single mutant. The period of the double mutant was however intermediate between the single mutants, being slightly reduced compared to the *soc1 *single mutant (τ = 24.49 ± 0.24 h vs. 24.98 ± 0.22 h, *P*= 0.048). The resulting period of *soc1; flc *was identical to that of Col (Figure 2 and 5, Table 2), but longer than that of the *flc *null mutant (Figure 1). This result suggests that *FLC *may increase circadian period in part by repressing *SOC1*, but its effect also occurs by other means, consistent with the function of *FLC *in the floral-repression pathway [17].

#### FLM and SVP

*FLC *is a member of a six-gene sub-family of the MADS class, based on sequence similarity. *FLOWERING LOCUS M *(*FLM*) (also known as *MAF1/AGL27*), is the member of this family with the highest level of identity with *FLC *[34]. Over-expression studies have shown *FLM *to have a similar function in the repression of flowering as *FLC*. However, unlike *FLC*, *FLM *expression is not increased by *FRI*, and *FLM *does not contribute to the vernalization requirement [34,35]. Leaf-movement assays on the *flm *mutant revealed that it had no circadian defect (*P *= 0.69) (Figure 2 and 5, Table 2).*FLM *functions with another MADS box gene, *SVP *(*SHORT VEGETATIVE PHASE*) in repressing flowering time [35,36]. When we tested for circadian period, the *svp *mutant showed a significant lengthening in period of 0.7 h compared to wild type (τ = 25.15 ± 0.25 h vs. 24.44 ± 0.17 h, *P *= 0.006) (Figure 2 and 5, Table 2), in contrast to the significantly shortened circadian period of the *flc *mutant. These results indicate that *FLC *may have a unique function in shortening the circadian period of Arabidopsis, which is not shared by other MADS box transcription factors that function in the flowering-time pathways.

### Seasonal regulation of the circadian clock by vernalization

**Figure 3:**
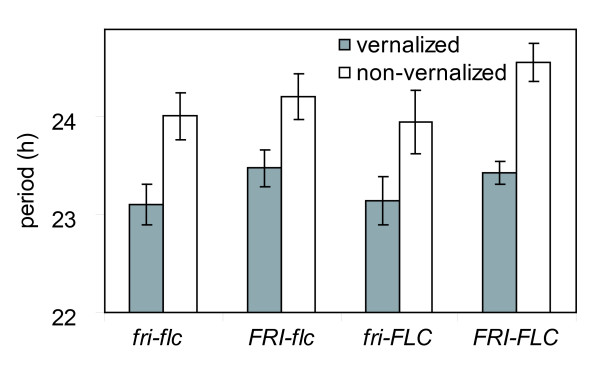
**Effects of vernalization on the Arabidopsis clock **Comparison of circadian periods of vernalized and non-vernalized Arabidopsis mutant seedlings harboring all possible combinations of *FRI-FLC *(*n *= 18–26). After vernalization treatment at 2°C for 8 weeks, plants were assayed for rhythmic leaf movement for approx. 1 week in constant dim white light. vern *fri-flc *τ = 23.10 ± 0.21 [SEM] h, non-vern *fri-flc *τ = 24.01 ± 0.24 h, vern *FRI-flc *τ = 23.48 ± 0.19 h, non-vern *FRI-flc *τ = 24.21 ± 0.23 h, vern *fri-FLC *τ = 23.14 ± 0.25 h, non-vern *fri-FLC *τ = 23.95 ± 0.32 h, vern *FRI-FLC *τ = 23.43 ± 0.12 h, non-vern *FRI-FLC *τ = 24.56 ± 0.19 h.

*FLC *expression represses flowering in biennial and winter-annual plants, causing overwintering in the vegetative state. Vernalization relieves the floral repression in part by stably suppressing *FLC *expression, so flowering can proceed in the Spring [37]. *flc *mutants retain some vernalization response [17], which may be mediated by other MADS-box genes [38]. Based on the prediction that any effect on *FLC*-expression levels, whether genetic or physiological, should alter circadian period, we sought to test the effect of vernalization on circadian period and the effect of *flc *mutations on the clock after vernalization. We assayed circadian period in seedlings harboring the *FRI *and *FLC *alleles, as described above, comparing seedlings that had been vernalized to controls grown without vernalization. Flowering-time analysis of these plants, following the leaf movement assays, confirmed the effectiveness of the vernalization treatments in accelerating flowering (data not shown). We found in duplicate experiments that vernalization consistently decreased circadian period (*P *< 0.001) but that none of the single-gene or gene interaction effects was significant (Figure 3, Tables 3 and 4). Therefore, vernalization consistently shortened circadian period, regardless of *FLC*. There is a possibility that development during the vernalization period may have caused the change in circadian period. However, prior to these experiments, we identified growth conditions for the vernalized and non-vernalized plants such that in these experiments, seedlings of both groups were phenotypically indistinguishable from one another. Furthermore, as the same primary leaf pair was assayed in both treatment groups, a directly comparable developmental trait comparison was made (see Materials and Methods).

## Discussion

We report here that the genes of the autonomous floral-promotion pathway, and *FLC *itself, modulate the period of a circadian clock in Arabidopsis. The effects on period are modest, but our data measurements are accurate, as detected in plots of relative amplitude error (RAE) versus period length for individual cotyledon traces of a genotype in an experiment (see Additional file 1). Additionally, there is no noticeable increase in arrhythmic mutant plants relative to wild-type (data not shown). Data for all genotypes tested in this study were collected from multiple experiments and in each case the period effects relative to the wild type were consistent. The only exception to this was the *fpa *mutant, which had first a long period and then a short period, relative to wild-type, in two independent experiments. As expected from this result, statistical analysis of these data was unable to assign a function for *FPA *in the circadian clock. It should also be mentioned that in each of the multiple *FRI;FLC *mutant combination studies (Figure 1A, Table 1), *fri-flc *double mutant period was always reduced compared to *FRI-FLC *period. Direct comparison with other genotypes between replicate experiments was not reasonable without the statistical analysis we performed using REML.

The mechanisms by which the floral-promotion genes affect circadian period are similar, but not identical, to their control of flowering time. Regulators other than *FLC *must be involved because, for example, *LD *and *FVE *affect the clock significantly in the *flc *mutant background. We also reveal a vernalization-dependent shortening of the circadian period. Recent studies in chestnut seedlings have shown that during the chilling period, circadian expression of genes homologous to Arabidopsis core-clock genes are suppressed, with cyclic expression of these genes resumes post-chilling [39]. Our studies however identify alterations in the Arabidopsis clock after the cold-exposure period. *FLC *would be an obvious candidate gene to mediate this response, however, our studies did not fully support this possibility. Circadian analysis of mutants in other vernalization-responsive MADS-box genes, such as *MAF2 *[34,40] may shed light on the how the clock is altered by vernalization. It is unclear which components of the circadian-clock mechanism are the targets that mediate these period changes. Rhythmic, transcriptional-translational feedback loops are important in circadian timing and the genes tested here are regulators of gene expression, though *FLC *is not thought to be rhythmically regulated [41]. It is possible that the expression level of one of the clock genes is *FLC*-dependent, for example. Given the modest effects we observed upon circadian period, the *FLC*-dependent change in expression level might be very slight.

From an ecological perspective, the stable effects of vernalization allow plants to distinguish between Spring and Autumn, even though both seasons have an equal day length. Our work suggests that an additional mechanism may contribute to this distinction, namely that the circadian clocks of plants run "faster" in Spring than in Autumn.

Environmental changes can thus have "after-effects" on the circadian clock, in Arabidopsis, as in other organisms. These are typically observed as an alteration in the circadian period immediately after exposure to exotic light-dark or warm-cold cycles [11,42]. The period-shortening effect of vernalization is expected to be much longer-lasting. The physiological consequences of the period shortening will depend on which rhythms are affected, and how the change in period under constant conditions affects the rhythms under day-night cycles. Short-period mutations can reduce the critical photoperiod in Arabidopsis, leading to earlier flowering under shorter photoperiods [43]. If the shortened circadian periods due to *FLC *repression affected rhythmic *CO *expression, this would reinforce the acceleration of flowering in Spring days compared to Autumn days, by induction through the photoperiod pathway, in addition to the removal of autonomous-pathway flowering repression. Such cross-talk between the autonomous pathway and the circadian system emphasizes the networked structure of plant-signaling circuits. These include the circuits adapted to mediate plastic responses to rhythmic, daily, and seasonal environmental signals.

## Conclusion

**Figure 4:**
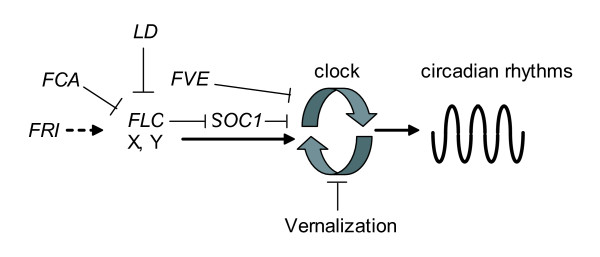
**Schematic of *FLC*'s role in the Arabidopsis circadian clock. **A schematic of *FLC*'s role in regulating circadian period. *FLC *function increases circadian period (arrow). This function is marginally enhanced by *FRI *(dashed arrow) and perhaps mediated in part by repressing (blunt arrow) *SOC1*, which decreases period (blunt arrow). Flowering-time genes of the autonomous pathway such as *LD *and *FCA *maintain shorter circadian periods in part by repressing *FLC *but with significant contributions via other components (X and Y, drawn with *FLC *for clarity only). Vernalization also regulates circadian period by an unidentified mechanism. Not all genes of the autonomous flowering pathway influence circadian timing (e.g. *FPA*, not shown).

**Figure 5:**
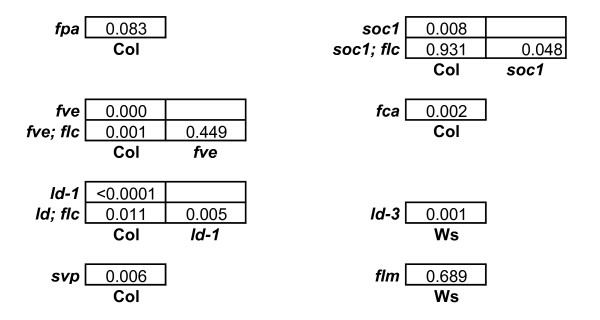
**Statistical significance testing for circadian period effects of floral pathway mutant lines. **Measures of significance for comparisons among circadian periods from Table 2, calculated from the standard errors of the differences.

We demonstrated that several genes in the Arabidopsis autonomous-flowering pathway are also involved in regulation of the circadian clock. We identify *FLC *as a dose-dependant regulator of circadian period and identify autonomous-pathway genes regulating the clock in both an *FLC *dependant and independent manner. As *FLC *expression levels are reduced by vernalization, we tested the hypothesis that circadian period was altered in vernalized *FLC *wild-type plants. Though we could not firmly establish *FLC *as the mediator of vernalization's effect on circadian period, we showed conclusively that vernalization alters circadian period in Arabidopsis. Figure 4 is an illustrated schematic of how vernalization and the genes we tested may be regulating circadian period in Arabidopsis.

## Methods

### Plant materials

The following mutant lines in the Columbia-0 background have been described previously:

*FRI-Sf2;FLC-Col, FRI-Sf2;flc-3, fri*-*Col;FLC-Col, fri-Col;flc-3*, 35S:*FLC *[16]; *soc1-2, soc1-2;flc-3 *(previously *agl20, agl20;flc-3*) [44];*fve-4, fve-4;flc-3 *[45];*ld-1, ld-1;flc-3, fpa-7 *[17]. Mutants in the Ws-2 background have been described: *flm *[46], *ld-3 *[24]. We are grateful for seed of *fca-9 *[47] from Caroline Dean (Norwich), of *svp-41 *[36] from Peter Huijser (Cologne), and of *ld-1 *from the Nottingham Arabidopsis Stock Centre.

### Growth and imaging conditions

Seedlings were grown and followed by assays of rhythmic leaf movements by video imaging, as described by Dowson-Day and Millar, 1999; Millar *et al*. 1995 [48,49]. Briefly, surface-sterilized seeds were plated on 1.5% Murashige-Skoog agar medium plates [50] that included 3% sucrose, and were stratified for 3 days at 4°C. Seedlings were germinated under 30–40 μmolm^-2^sec^-1 ^continuous cool white fluorescent light for 7 days, at 21–22°C, followed by entrainment to three 12 h-light, 12 h-dark cycles. Growth of the first pair of primary leaves was recorded under 30–40 μmolm^-2^sec^-1 ^continuous cool white fluorescent light, at 21–22°C for 7 days. Seedlings were arranged randomly with respect to genotype within each experiment, to avoid positional bias in the imaging arrays. For vernalization, stratified seed were germinated for 4 days, as described above (at which point cotyledons were expanding), then incubated at 2°C for 8 weeks under low-intensity white light (0.5–1.0 μmol m^-2^sec^-1^). After the vernalization treatment, seedlings were transferred to continuous light for 4 days, then entrained and imaged as above. In order to confirm the effectiveness of vernalization, imaged seedlings were transferred from agar to soil immediately after the leaf-movement assay. Flowering time of these lines was measured as the number of rosette leaves when the floral bolt was 1 cm high. These studies confirmed the expected effects of *FLC *and *FRI *upon vernalization-responsiveness (data not shown).

### Data analysis

Leaf movement data were analyzed by Fast Fourier Transform non-linear least squares program FFT-NLLS [51], essentially as described in Dowson-Day and Millar, 1999 [48]. The circadian period of each genotype was estimated as the variance-weighted mean of the most significant period within the circadian range (15–35 h) for each leaf. In order to make the most efficient use of data gathered in separate experiments, the data from all the experiments were analyzed jointly using REML [52] in the statistical package GENSTAT 5 [53]. REML is a generalization of analysis of variance and is appropriate for the analysis of unbalanced data. Genotypes to be compared directly were included in the same experiments. In the analyses, experiment, camera within experiment, plant within camera, and cotyledon within plant were taken as random factors, with mutant line as a fixed factor. The analysis was weighted to allow for the inherent variabilities of estimation of period from the different traces. The period estimate for each leaf recording was weighted for analysis by the reciprocal of the error associated with the period, as estimated by FFT-NLLS. Significance of *FRI-FLC *interactions and vernalization effects on period were assessed using the Wald test with variances derived from REML. The significance of the differences between the mean period of pairs of genotypes was assessed using the standard error of each difference, derived from REML. Figures 1, 2,3 report the conventional SE of each genotype mean.

## Authors' contributions

NS, AJM, and SJD conceived the experiments and wrote the paper, with critical experimental and intellectual revisions from SDM and RMA. SDM and RMA generated reagents. NS carried out the experiments. JRL conducted statistical analysis.

## Supplementary Material

Additional file 1 **Robustness of rhythmicity of all lines examined **MS EXCEL file containing 3 worksheets. These include RAE plots for all genotypes examined (sorted with respect to Figure 1, 2, 3 in the main article body). Click here for file

## Figures and Tables

**Table 1:** *FLC *effects on circadian period.

genotype	**fri;flc**	**FRI;flc**	**fri;FLC**	**FRI;FLC**	**Col**	**35S:FLC**
period(h)	24.07	24.00	24.40	24.90	24.44	25.65
s.e.m	0.31	0.32	0.29	0.31	0.17	0.31
n	85	82	107	87	155	36
P	_	_	*0.004	*0.004	_	** < 0.0001

Mean circadian periods of leaf movement in Arabidopsis mutant and wild-type seedlings, calculated using REML analysis.

* *P *from Wald test; indicates the statistical significance of *FLC*'s effect alone, by comparison of *FLC *to *flc *irrespective of *FRI/fri*.

** P from the standard error of the difference; indicates the statistical significance of 35S:*FLC *effect, compared to Col wild-type.

s.e.m., standard error of the mean.

n, number of contributing leaf traces.

**Table 2:** Circadian period of floral pathway mutant lines.

genotype	**Col**	***fpa***	***soc1***	***soc1;flc***	***fve***	***fve;flc***	***fca***
period(h)	24.44	24.91	24.98	24.49	25.54	25.30	25.28
s.e.m	0.17	0.27	0.22	0.24	0.30	0.27	0.28
n	155	46	69	70	36	36	39
							
genotype	**Col**	***ld-1***	***ld;flc***		**Ws**	***ld-3***	

period(h)	24.44	25.93	25.12		24.15	25.51	
s.e.m	0.17	0.25	0.27		0.26	0.39	
n	155	53	43		49	19	
							
genotype	**Ws**	***flm***			**Col**	***svp***	

period(h)	24.15	24.13			24.44	25.15	
s.e.m	0.26	0.35			0.17	0.25	
n	49	25			155	53	

Mean circadian periods of Arabidopsis mutant and wild-type seedlings, tested as in Figure 1.

**Table 3:** Circadian period of lines pre- and post-vernalization.

Vernalized plants				
genotype	***fri;flc***	***FRI;flc***	***fri;FLC***	***FRI;FLC***
**period(h)**	**23.10**	**23.48**	**23.14**	**23.43**
s.e.	0.21	0.19	0.25	0.12
n	20	24	20	26
				
**Non-vernalized plants**				
genotype	***fri;flc***	***FRI;flc***	***fri;FLC***	***FRI;FLC***

**period(h)**	**24.01**	**24.21**	**23.95**	**24.56**
s.e.	0.24	0.23	0.32	0.19
n	22	18	23	24

Mean circadian period of Arabidopsis mutant seedlings, tested as in Figure 1, with or without vernalization at 2°C for 8 weeks.

**Table 4:** Statistical significance testing for period effects of vernalization.

Gene factor × vernalization	*P
**vernalization(minus FRI, FLC factors)**	**<0.001**
*FRI*	0.842
*FLC*	0.692
*FRI × FLC*	0.461

* *P *from Wald test; indicates the statistical significance of vernalization treatment effects alone and vernalization × gene effects on circadian period.
